# Coeliac disease in infants: antibodies to deamidated gliadin peptide come first!

**DOI:** 10.1186/s13052-017-0392-6

**Published:** 2017-08-10

**Authors:** Michele Arigliani, Francesca Rech Morassutti, Martina Fabris, Paola Melli, Elio Tonutti, Paola Cogo

**Affiliations:** 1 Department of Clinical and Experimental Medical Sciences, Unit of Pediatrics, University Hospital of Udine, Piazzale S. Maria Misericordia 1, 33100 Udine, Italy; 2 Department of Laboratory Medicine, Institute of Clinical Pathology, University Hospital of Udine, Udine, Italy

**Keywords:** Coeliac disease, Anti-deamidated gliadin peptides antibodies

## Abstract

**Background:**

The onset of coeliac disease (CD) in the first year of life is uncommon and the diagnosis can be challenging due to the suboptimal sensitivity of tissue transglutaminase antibodies (tTG) at this age and the many other possible causes of malabsorption in infants. Antibodies to deamidated gliadin peptides (anti-DGPs), especially IgG, may appear earlier than IgA anti-tTG in very young children with CD.

**Case presentation:**

We report here on an 8-month-old child who was evaluated for failure to thrive, constipation and developmental delay. The symptoms started following gluten introduction in the diet. Laboratory tests showed high fecal elastase concentration, normal serum IgA levels with positive IgG and IgA anti-DGPs, whereas anti-tTG were not detected. The duodenal biopsy revealed a complete villous atrophy (Marsh-Oberhuber 3C). The culture of biopsy fragments in the presence of gliadin peptides did not stimulate the production of IgA anti-endomysial antibodies. Genetic testing proved the child was positive for HLA-DQ2 (DQA1*05; DQB1*02) and HLA-DQ8 (DQA1*03, DQB1*0302). Having initiated the gluten-free diet, the symptoms disappeared and the infant experienced rapid catch-up growth with normalization of psychomotor development.

**Conclusions:**

This case report highlights the utility of anti-DGPs for screening infants with suspected CD. The pattern with positivity for IgG and IgA anti-DGPs only is rare in IgA-competent children with biopsy-proven CD. It could be explained in infancy as immaturity of the adaptive immune system.

## Background

Coeliac disease (CD) is an autoimmune disorder triggered by gluten ingestion in genetically predisposed subjects [1]. While it is know to affect around 1% of Caucasian schoolchildren, the true prevalence is probably underestimated [2, 3]. While coeliac disease can occur at any age following gluten introduction in the diet, the onset of symptoms shortly after weaning is uncommon [4–6]. The pathogenesis of the disease depends on the presence of gliadin-reactive CD4+ T cells in the *lamina propria* of the small bowel, which recognize gliadin peptides deamidated by tissue transglutaminase and bound to DQ2+ or DQ8+ antigen-presenting cells [7, 8]. The gliadine-reactive CD4+ T cells enhance an adaptive immune response that leads to intraepithelial and *lamina propria* infiltration of inflammatory cells, crypt hyperplasia, and villous atrophy [9]. Innate immunity also contributes to mucosal damage [10]. Tissue transglutaminase antibodies (tTG) are directed against the enzyme responsible for the deamidation of gliadin in the *lamina propria*. These antibodies perform at a lower sensitivity and specificity in children under 18 months of age compared to older subjects, the earliest tTG seropositivity being reported at the age of 12 months [11–14]. Tests for antibodies to deamidated gliadin peptides (anti-DGPs) have replaced those for anti-gliadin antibodies, as the former show higher specificity [15]. Immunoglobulin G anti-DGPs are more sensitive than IgA in the diagnosis of CD [16–19]. The combination of IgA anti-tTG and IgG anti-DGP offers the best accuracy for diagnosis of CD at all ages, as it increases the chances of detecting the disease in subjects with IgA deficiency [17, 20–23]. Moreover, the presence of IgG anti-DGPs seems to be the best serologic marker of villous atrophy in the follow up of coeliac patients [24–26]. While in older patients IgA anti-tTG levels generally correlate with the severity of the duodenal lesions according to the Marsh–Oberhuber grading system [27, 28], in young children high IgG anti-DGP titres are related to severe intestinal damage [29–32].

In this article we present the unusual case of an infant who developed symptoms of CD shortly after weaning. In spite of a complete villous atrophy at biopsy, only serum anti-DGPs were increased, whereas anti-tTG and anti-endomysial antibodies were absent.

## Case presentation

An 8-month-old boy was evaluated for failure to thrive and developmental delay. He was born at 38 weeks of gestational age (birth weight 4.090 kg, above the 90th percentile). Familial and antenatal history was unremarkable; in particular there was no history of gestational diabetes nor any significant perinatal event. The patient had been exclusively breastfed for the first 5 months of life, with regular growth and psychomotor development. After weaning, at between 6 and 8 months of age, the child experienced worsening constipation, bulky stools, irritability and anorexia. The onset of symptoms had no association with any specific event. The infant was admitted to the pediatric ward of the University Hospital of Udine, Italy, when he was 8-months old. His weight-for-length was below the 3rd percentile, whereas 2 months before it was at the 25th percentile [33]. Between the 6th and 8th months he also showed developmental regression: his babbling and smiling decreased, he could no longer sit without support and became apathetic and irritable. The head circumference had increased normally since birth at around the 25th percentile. The physical examination revealed severe dystrophy, with a potbelly and wasted limbs. He appeared apathetic with little interest in things and people around him. He also had mild hypotonia, with decreased muscle bulk but preserved deep tendon reflexes. Laboratory tests revealed a normal full blood count. A metabolic panel showed serum albumin (3.6 g/dL;), Vitamin A (207 mcg/L) and total IgG (269 mg/dL) levels at the lower limit of normal. A screening for CD revealed normal IgA levels, absence of tTG antibodies but positive serum anti-DGPs (IgG 183 U/mL; IgA 147 U/mL; normal range < 10 U/ml for both, as suggested by the manufacturer). Tissue-tranglutaminase antibodies and anti-DGPs were assessed by a chemiluminescence enzyme immunoassay (Zenit-RA®, Menarini Diagnostics, Florence, Italy). All other laboratory tests of the metabolic panel were normal (including iron levels, folic acid, vitamin D, B12, and E, thyroid-stimulating hormone, creatinine kinase, venous blood gas, lactate and ammonia levels). Fecal elastase was at a low concentration (<100 mcg/g), while fecal calprotectin concentration (IDK® Calprotectin ELISA, Immundiagnostik, Bensheim, Germany) of 135 mg/kg was in the normal range for the patient’s age [34, 35]. A normal sweat chloride test ruled out cystic fibrosis and a magnetic resonance imaging of the brain excluded structural/anatomic brain abnormalities.

An esophagogastroduodenoscopy with duodenal biopsy showed complete villous atrophy with crypt hypertrophy and more than 60 intraepithelial lymphocytes per 100 enterocytes in the duodenal mucosal, corresponding to a 3C score of the Modified Marsh (Oberhuber) classification. Antiendomysial IgA antibodies were not detected in the supernatant of duodenal mucosal fragments cultured with gliadin peptides [36]. Genetic analysis revealed the presence of HLA-DQ2 (DQA1*05; DQB1*02) and HLA-DQ8 (DQA1*03; DQB1*0302). Within 4 months of initiating a gluten-free diet, the child caught up with his original weight-for-age z-score, with complete normalization of neurological evaluation and laboratory parameters. Six months after the diagnosis, IgG anti-DGP were still slightly positive (20 UI/ml, normal range < 10 UI/ml), while IgA anti-tTG were absent. After 6 months and for the next 2 years both DGP and tTG antibodies resulted negative.

## Discussion

In this case report, the serological pattern with DGP-positive/tTG-negative antibodies in an infant was predictive of CD with villous atrophy. The finding confirms evidence from literature that anti-DGPs are a sensitive marker of CD in very young children and may be the first CD antibodies to seroconvert [37, 38]. This would be in accordance with the suggested pathogenesis of CD, as the disease process seems to initiate with the T-cell responses to deamidated gliadin peptides in the intestinal mucosa [39]. However, only a minority of children with circulating T cells specific to deamidated gliadin will develop anti-DGPs and celiac disease. Peripheral blood T-cell responses to deamidated gliadin peptides were detectable at 9 months of age in 40% of a cohort including 300 children with HLA-associated genetic risk for CD. However only 3.1% of the cohort’s subjects had developed CD by the age of 4 years, each being seropositive for IgG anti-DGPs and IgA anti-tTG at the time of diagnosis [40].

Lammi et al. showed that in 35 of the 48 children with CD from the Finnish DIPP study [41], serum IgG anti-DGPs preceded tTG positivity and appeared on average 1 year earlier [19]. Our patient, unlike the children with anti-DGPs seropositivity and CD described by Lammi et al. [19], never developed tTG antibodies in spite of severe gluten-sensitive enteropathy.

The positivity of serum anti-DGPs only in very young children has a poor positive predictive value for CD and should be considered with caution: in a retrospective analysis, Parizade found that over 60% of children under 2 years of age with DGP-positive/tTG-negative pattern (HLA alleles not reported) became seronegative within 1 year, without following a gluten-free diet [42]. In that study, 6 out of 12 children with only anti-DGP positivity referred for biopsy, had a diagnosis of CD [42]. In the study by Olen and colleagues, [43] villous atrophy was found only in 8 of 149 children with DGP-positivity and tTG-negativity. These findings would suggest duodenal biopsy may be indicated in infants with sole anti-DGP positivity only if they have signs/symptoms consistent with CD and positivity for HLA DQ2/DQ8, as was the case in our patient [42, 44].

The children reported here was at standard genetic risk of CD, given the presence of the HLA-DQ2 (DQA1*05; DQB1*02) and HLA-DQ8 (DQA1*03, DQB1*0302) [4], while in a case-control study by Megiorni et al. [45], the HLA haplotype with copresence of DQ2 and DQ8 resulted at the top of the genetic risk gradient for CD (risk 1:7).

The infant’s fecal elastase was low, with chronic malabsorption. In subjects with villous atrophy, the mucosal damage may impair the release of enteric hormones (secretin and cholecystokinin) and cause secondary reduction of exocrine pancreatic secretion, which includes fecal elastase [46–50]. However in children with CD, fecal elastase concentration usually normalizes after a few months from the introduction of a gluten free diet [51], as in the case presented here.

The patient described in this paper had neurological symptoms at presentation, such as developmental delay and hypotonia. These symptoms are sometimes reported in children with onset of CD during infancy, possibly due to vitamin and micronutrient deficiency [52]. In our patient, no such nutritional deficit was identified although zinc levels, which are linked to neurocognitive delay in malnourished children, could not be investigated [53].

Timing of gluten introduction in our patient was in accordance with current international recommendations [37] and could not influence the onset of CD. However, recent evidence indicates that neither early (<17 weeks of life) nor delayed gluten introduction (>26 weeks) is a risk factor for future CD [54].

## Conclusions

This case report highlights the utility of IgG anti-DGPs for screening infants with suspected coeliac disease. Some of the children who develop CD at this age, might have an immature adaptive immune response with the sole production of anti-DGPs even in the presence of villous atrophy, as was the case in our patient. We suggest that the serologic pattern with DGP-positive/tTG-negative antibodies in an infant with consistent symptoms and HLA DQ2/DQ8 haplotype should prompt a duodenal biopsy to rule out CD. Further studies are needed to provide a more detailed evaluation of this serologic pattern in IgA-competent children with celiac disease.

## Abbreviations

CD: Coeliac disease
DGPs: Deamidated gliadin peptides
HLA: Human leukocytes antigen
tTG: Transglutaminase antibodies
